# Sparse Damage Detection with Complex Group Lasso and Adaptive Complex Group Lasso

**DOI:** 10.3390/s22082978

**Published:** 2022-04-13

**Authors:** Vasileios Dimopoulos, Wim Desmet, Elke Deckers

**Affiliations:** 1 Department of Mechanical Engineering, KU Leuven, Celestijnenlaan 300, 3001 Leuven, Belgium; wim.desmet@kuleuven.be; 2 DMMS Lab, Flanders Make, 3001 Leuven, Belgium; elke.deckers@kuleuven.be; 3 Department of Mechanical Engineering, KU Leuven, Wetenschapspark 27, 3590 Diepenbeek, Belgium

**Keywords:** sparse damage detection, complex Group Lasso, adaptive complex Group Lasso, low-frequency inspection

## Abstract

Sparsity-based methods have recently come to the foreground of damage detection applications posing a robust and efficient alternative for traditional approaches. At the same time, low-frequency inspection is known to enable global monitoring with waves propagating over large distances. In this paper, a single sensor complex Group Lasso methodology for the problem of structural defect localization by means of compressive sensing and complex low-frequency response functions is presented. The complex Group Lasso methodology is evaluated on composite plates with induced scatterers. An adaptive setting of the methodology is also proposed to further enhance resolution. Results from both approaches are compared with a full-array, super-resolution MUSIC technique of the same signal model. Both algorithms are shown to demonstrate high and competitive performance.

## 1. Introduction

Rytter [1] divided the problem of damage identification into the four fundamental levels of detection [2], localization [3], quantification [4] and prediction [5]. An inspector would acquire precise knowledge for each of these steps with information characteristic for the system. For many years, the latter was an obstacle in the process of damage detection as it would often necessitate numerous and time-consuming experiments. Nevertheless, recently researchers have been focusing on compressive sensing to offer a solution. In compressive sensing, one seeks to obtain critical information about the state of a system while relying on limited measurements or experiments. As a result, compressive sensing algorithms are often able to offer an efficient alternative to traditional full-array techniques for damage identification. On the other hand, compressive sensing naturally faces the obstacle of underdetermination since the number of unknown parameters significantly exceeds that of observations.

An important subset of underdetermined problems consists of the cases where the unknown variables and measured observations are connected through linear relationships. These underdetermined linear problems, which frequently occur in engineering, are traditionally treated by means of the Ordinary Least Squares (OLS) approach [6]. However, the solutions obtained with this approach are in general known to be non-unique. Therefore, any predictions made are rarely meaningful. As an alternative, regularization techniques such as the Ridge regression [7] introduce a penalty term in the OLS cost function and balance between estimation’s bias and variance while also favoring certain solutions. More precisely, in Ridge regression, a penalty is introduced as the $l_{2}$-norm of the solution vector scaled by a tuning parameter. Employing this minimization problem leads the second norm penalty to shrink all coefficients by the same factor. As a result, the solution model is often large with many variables being selected, a problem also seen with OLS. Tibshirani [8], with the Least Absolute Shrinkage and Selection Operator (Lasso), introduced the $l_{1}$-norm as an alternative penalty for the regression problem. In contrast to the second norm, the $l_{1}$ penalty minimizes the cost function while it also promotes sparse predictions. This is of significance considering it is often the case that the nature of the solution is sparse over the exploration space. In other words, this is a solution where the majority of unknown variables remain inactive. Audio signal processing [9], Magnetic Resonance Imaging (MRI) [10] and genomic selection [11] are characteristic examples for this family of problems. Similarly, damage, being the topic of this paper, tends to be quite a localized phenomenon that possesses a sparse behavior. Namely, considering a set of possible damage locations, it is reasonable to assume that only a small number of them are actual points of degradation for a real damage case. Therefore, it would make sense to include a sparsity penalty in the solution process and approach damage detection by means of a sparsity constrained routine.

To this end, Smith et al. [12] proposed a sensitivity-based approach for sparse damage detection. In their work, they demonstrated that small deviations for the impulse responses between the damaged structure and its counterpart baseline can be approximated through the linear combination of sensitivity vectors pointing to the sites of defects. As a result, they were able to define a linear regression problem. The solution of this approximation with Lasso indicated the location of damage. One should note that such an approach calls for a sufficiently accurate model for the sensitivities of the baseline state to be obtained. Chen et al. [13] applied a sensitivity analysis to express shifts of the modal parameters in terms of local stiffness degradation on the elemental level of a structural model. Sparse regression was then solved with a sequential threshold least squares algorithm. In addition, they introduced an $l_{2}$ Bayesian method to update the baseline model and quantify uncertainty before the regression was applied. Sensitivity analysis using modal data was also implemented by Hou et al. [14]. In this work, a regression problem was solved using the elastic net method [15]. Following this approach, the authors were able to simultaneously introduce the benefits of $l_{1}$-norm and $l_{2}$-norm regularization in damage detection. Using a similar sensitivity framework, Fan et al. [16] exploited sparsity for damage detection with the sensitivities of the resonance frequency shifts of the impedance responses. This technique was found to localize damage under various temperature conditions.

Furthermore, optimization methods have been used along with compressive sensing and damage detection to localize damage by solving the regression problem or by proposing optimal detection parameters. Hou et al. [17] introduced a genetic algorithm in the sensitivity framework that identifies optimal sensor placement for increased performance due to sensitivity matrices of least mutual coherence. Chen et al. [18] employed swarm intelligence in order to solve a sparsity constrained problem where the objective functions were defined as the change of modal parameters before and after the damage occurred. In their work, they also demonstrated the possibility for independent weighting between the different objectives for additional flexibility. Ding et al. [19] treated damage detection as an optimization problem and proposed an improved Jaya algorithm to solve the sensitivity-based objective functions. It was found for the introduced technique to outperform state-of-the-art optimization methods.

Sparse representation is another approach for compressive damage detection. This technique aims to decompose measured responses in terms of the elements in a given dictionary matrix. Yang and Nagarajaiah [20] introduced such a framework of sparse representation by assembling damage-dependent modal feature vectors to localize defects with sparsity-lead algorithms. Following a similar approach, Wang et al. [21] constructed dictionaries appropriate for the decomposition of received guided-wave responses into a spatial domain with sparsity-based optimization. Chen et al. [22] constructed dictionaries for sparse representation based on an empirical mode decomposition and sparse coding. The first approach utilized a basis of intrinsic mode functions while the latter followed an adaptive logic where both the dictionary and the solution are iteratively updated until convergence. Kong et al. [23] utilized self-similarity features of planetary drivetrain data to develop data-driven dictionaries for damage detection in planet bearings. The use of such dedicated dictionaries enhanced diagnosis accuracy and performance.

Wave–defect interactions due to guided waves propagation in thin structures [24] have also been used for sparse damage detection. Levine et al. [25] decomposed time-domain scattering signals with a simulated basis of responses emitted from assumed defects on different locations. The authors recognized that knowledge of the scattering pattern of the defect is needed and made the necessary approximations. Sen et al. [26] constructed a response library of scattered signals on a thin plate after placing a mass over a set of grid points. They then applied the Lasso regression for a scattered field of unknown damage location and the response library. This approach also suffers from regression sensitive to the assumed damage. Alguri et al. [27] used guided waves to localize defects when the decomposition basis was built using surrogate information.

Overall, methods such as the ones discussed above utilize time-domain response signals, modal frequencies, the system’s stiffness matrices and the Modal Assurance Criterion (MAC) to perform damage detection. In principle, this is all real-valued information. The reason being that standard Lasso is not suitable to operate on complex data. However, it is often the case that complex data are available in engineering analysis. For instance, by means of a frequency domain transformation, complex transfer functions offer a clear insight into the physics of the system. In addition, time-domain operations performed between signals are significantly simplified in the frequency domain. Namely, a convolution gets transformed to a much more intuitive linear multiplication in the frequency domain. This simplification and the inherent linearity are a strong drive for sparsity-based damage detection methods that utilize the complex frequency response functions (FRFs).

A basic implementation for this would be the trivial approach of performing the Lasso on the real and imaginary signal components independently. However, such an approach would ignore coupling phenomena expressed between the real and imaginary terms, resulting in an algorithm of degraded performance. A solution that assembles both real and imaginary entries in a single matrix was presented in the literature [28,29]. In both works, Lasso using complex data was shown to be equivalent to a Group Lasso algorithm with two groups of variables. The latter is an extension of standard Lasso which was introduced by Yuan [30] to solve the regression problem for cases where variables must be selected in groups.

In this paper, we present two sparsity-based methods that utilize low-frequency response functions for damage detection. More precisely, two complex Group Lasso strategies for the localization of multiple defects are proposed. The presented techniques are able to localize damage by means of a single sensor interrogating the structure’s vibration responses.

The rest of this paper is organized as follows. In Section 2, the signal model for the damage detection method is introduced. In Section 3, the methodologies for damage detection with the complex Group Lasso and an adaptive complex Group Lasso algorithm are presented. In Section 4, experimental campaigns are performed to evaluate the performance of both techniques for damage detection. In Section 5, the obtained results are compared with respect to the output of a full-array and a single sensor Multiple Signal Classification (MUSIC) algorithm which serves as a well-defined reference [31,32]. In Section 6, the influence of noise on the performance of the proposed damage detection techniques is evaluated. Section 7 concludes the paper and highlights its main contributions.

## 2. Signal Model

Consider an elastic medium with *L* embedded point-like defects and a sequence of *m* temporal Dirac excitations being applied on *m* points of the structure. Responses are then collected on a sensing element for each of the excitations. In a second experiment, the same process is repeated for a healthy baseline of the structure. Due to wave superposition and assuming linearity, the subtraction of these two sets of measurements isolates the scattering information emerging only from the *L* defects. These scattered signals can be expressed in the frequency domain by means of a Fourier transform.

After the transformation, assuming that the signals consist of *f* independent frequency components, we focus on a frequency bin $f_{i}$. Specifically, we construct a vector $H(f_{i})$ with elements the components of the *m* scattered signals at the bin $f_{i}$. According to Lev-Ari [33], the vector of coefficients $H(f_{i})$ is approximated by means of a matrix multiplication using the transfer functions of the background medium as

$$H\left(f_{i}\right)=T\left(f_{i}\right)\varrho \left(f_{i}\right)+\epsilon ,$$

(1) $$\left[\begin{array}{c} H_{1}\left(f_{i}\right)\\ H_{2}\left(f_{i}\right)\\ \vdots \\ H_{m}\left(f_{i}\right) \end{array}\right]=\left[\begin{array}{cccc} T_{11}\left(f_{i}\right) & T_{12}\left(f_{i}\right) & \cdots & T_{1l}\left(f_{i}\right)\\ T_{21}\left(f_{i}\right) & T_{22}\left(f_{i}\right) & \cdots & T_{2l}\left(f_{i}\right)\\ \vdots & \vdots & \ddots & \vdots \\ T_{m1}\left(f_{i}\right) & T_{m2}\left(f_{i}\right) & \cdots & T_{ml}\left(f_{i}\right) \end{array}\right]\left[\begin{array}{c} \varrho_{1}\left(f_{i}\right)\\ \varrho_{2}\left(f_{i}\right)\\ \vdots \\ \varrho_{l}\left(f_{i}\right) \end{array}\right]+{\left[\begin{array}{c} \epsilon_{1}\\ \epsilon_{2}\\ \vdots \\ \epsilon_{m} \end{array}\right]}_{.}$$

In this expression, terms $T_{xy}\left(f_{i}\right)$ serve as frequency components for transfer functions on the healthy background medium, after an excitation at point *x* and a response measured at point *y*. Assuming x=[1,2,…,m], the collection of *m* terms yields a vector of coefficients for signals measured at point *y*. This vector forms one of the columns for the matrix $T(f_{i})$. Then, by allowing the measurement point to vary with y=[1,2,…,l] and maintaining the same set of excitation points *x*, we form the remaining columns.

Under this assumption, we call the set *y* imaging points because in Equation (1) a vector on the *j*th column is explicitly associated with measurements performed on the *j*th point. Then, proceeding with the calculations, it is seen that this vector is multiplied with a coefficient $\varrho_{j}\left(f_{i}\right)$. According to [33], this coefficient represents the combined effect of a transfer function between point *j* and the sensor, multiplied by the scattering coefficient of a potential defect at point *j*. As a result, the value of $\varrho_{j}\left(f_{i}\right)$ constitutes a good metric to characterize the structural integrity at this position. Lastly, $\epsilon_{x}$ represents the additive white Gaussian noise being present during the experiment.

Equation (1) poses a linear problem of the general form y=Ax+b. For the solution, one would search for the unknown *x*, here being the combined scattering coefficients on the different imaging points. As it was indicated, a zero value for coefficient $\varrho_{j}\left(f_{i}\right)$ would mean a healthy point and that the respective *j*th column is not selected in the model, while a non-zero value would imply damage and the respective column to be of importance. Therefore, the *j*th point should be highlighted as a region of interest for possible damage if $\varrho_{j}\left(f_{i}\right)$ is non-zero.

When trying to solve this problem, one will notice that the number of unknown variables *l* in general exceeds that of independent observations *m*, hence the system is identified as underdetermined with no unique solution. Nevertheless, under the assumption that not all *l* points act as defects, it is reasonable to expect that any physically meaningful solution is sparse. That is, most elements of the solution vector ϱ should be zero-valued. Therefore, introducing sparsity-based algorithms should lead to reliable solutions for the regression problem of damage detection. Thus, the goal would be to implement a Lasso-based algorithm to solve Equation (1). The minimization problem reads as,

(2) $$L\left(\varrho \right)=min_{\varrho }\left\{\frac{1}{2}{∥\begin{array}{c} H-T\varrho \end{array}∥}_{2}^{2}+\lambda {∥\begin{array}{c} \varrho \end{array}∥}_{1}\right\}.$$

In Equation (2), the $l_{1}$-norm penalty promotes sparsity in the solution while λ denotes a regularization parameter which dictates the level of sparseness. Intercept terms of the linear problem are neglected for low energy zero-mean white Gaussian noise. In addition, the frequency subscripts of Equation (1) have been suppressed for the sake of simplicity. Nevertheless, an independent regression problem still applies for each frequency bin. This notation will be followed for the rest of the paper unless otherwise stated.

## 3. Sparse Damage Detection with Complex Group Lasso

As discussed, since Equation (2) operates in the frequency domain it has to account for complex data. For that reason, the transformation proposed by Carlin [28] is applied in this section to express the Lasso regression with complex data as an equivalent Group Lasso problem of real entries.

### 3.1. Complex Group Lasso

In general, the Group Lasso algorithm addresses the following minimization problem:

(3) $$L\left(x\right)=min_{x}\left\{\frac{1}{2}{∥\begin{array}{c} Y-Ax \end{array}∥}_{2}^{2}+\lambda {∥\begin{array}{c} x \end{array}∥}_{2,1}\right\}.$$

In contrast to the $l_{1}$-norm penalty used during the Lasso regularization, the $l_{2,1}$-norm being present here does not only promote sparsity for the solution but also introduces a grouping effect. That is, variables are selected in groups to build the approximation.

Returning to the case of Equation (2), it is expected that once a complex coefficient is to become zero-valued, then both its real and imaginary components should simultaneously become zero-valued. Therefore, two groups of variables naturally form, one of real and one of imaginary terms. Equation (2) is thus written with respect to those two groups as

(4) $$L\left(\varrho \right)=min_{\varrho }\left\{\frac{1}{2}{∥\begin{array}{c} \left[\begin{array}{c} H_{r}\\ H_{i} \end{array}\right]-\left[\begin{array}{cc} T_{r} & -T_{i}\\ T_{i} & T_{r} \end{array}\right]\left[\begin{array}{c} \varrho_{r}\\ \varrho_{i} \end{array}\right] \end{array}∥}_{2}^{2}+\lambda {∥\begin{array}{c} \varrho \end{array}∥}_{1}\right\},$$

where subscripts *r* and *i* denote matrices of real and imaginary components, respectively. In Equation (4), the $l_{1}$-norm of complex components can also be written as

(5) $$\begin{array}{c} {∥\begin{array}{c} \varrho \end{array}∥}_{1}=\sum_{i=1}^{l}\left|\begin{array}{c} \varrho_{i} \end{array}\right|=\sum_{i=1}^{l}\sqrt{Re{\left(\varrho_{i}\right)}^{2}+Im{\left(\varrho_{i}\right)}^{2}} \end{array}=\sum_{i=1}^{l}{\left|\begin{array}{c} \varrho_{i} \end{array}\right|}_{2}={\left|\begin{array}{c} \tilde{\varrho } \end{array}\right|}_{2,1}.$$

The *l* rows of matrix $\tilde{\varrho }$ are populated with the pairs of real and imaginary components for the different $\varrho_{i}$. As a consequence of Equations (4) and (5), the complex minimization problem of Equation (2) is expressed as the following real-valued Group Lasso regression:

(6) $$L\left(\tilde{\varrho }\right)=min_{\tilde{\varrho }}\left\{\frac{1}{2}{∥\begin{array}{c} \left[\begin{array}{c} H_{r}\\ H_{i} \end{array}\right]-\left[\begin{array}{cc} T_{r} & -T_{i}\\ T_{i} & T_{r} \end{array}\right]\left[\begin{array}{c} \varrho_{r}\\ \varrho_{i} \end{array}\right] \end{array}∥}_{2}^{2}+\lambda {∥\begin{array}{c} \tilde{\varrho } \end{array}∥}_{2,1}\right\}.$$

In Equation (6), for known response vectors and matrices of transfer functions, a solution $\tilde{\varrho }$ is found. This solution, while being sparse, also accounts for the desired grouping effect. The implementation of such a minimization routine for damage detection is given in Algorithm 1. In this work, the formulated sparse regression problems are solved for each frequency bin in MATLAB using the SPAMS toolbox [34].

A Broadband Damage Indicator, BDI, is returned from the algorithm as the sum of unsigned regression coefficients across the frequency bins for each imaging point. Values of BDI are then assigned to the respective points’ coordinates to create a damage map across the structure. Peaks on the map indicate positions of damage.
**Algorithm 1:**Complex Group Lasso For Damage Detection
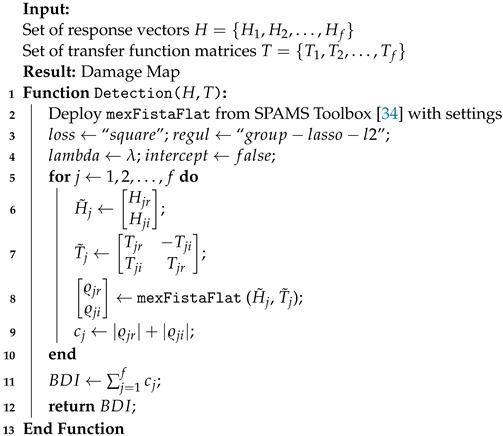


### 3.2. Adaptive Complex Group Lasso

As with Lasso, the accuracy of a solution in Equation (6) is subject to an appropriate selection for the regularization parameter λ. In that regard, Leng et al. [35] demonstrated that the Lasso selection model could be inconsistent. Specifically, they proved that with a probability greater than zero the true variables may not be included in the whole Lasso path of regularization parameters. Hence, they deemed Lasso not to possess the oracle property [36]. To this end, Zou [37] introduced an adaptive Lasso as an extension of regular Lasso which enjoyed oracle properties. The novelty of this development was to enable variable dependent regularization parameters. Therefore, instead of a common parameter for all variables, each one is scaled by a different factor. This process results in smaller and more accurate prediction models. In accordance, since the Group Lasso penalizes the regression coefficients in the same manner as Lasso, it should be expected that it suffers from the same inefficiency [38].

Nevertheless, in order for the adaptive Lasso to gain the oracle properties, a zero consistent initial estimate is necessary. In his work, Zou [37] proposed the Ordinary Least Squares (OLS) estimate as a zero consistent initial prediction. However, when the number of variables is too large the OLS estimate could be far from the truth. To that end, Wang [39] showed that the Group Lasso estimate can also be used as the initial guess, especially for cases where variables exceed in number the observations.

**Figure 1:**
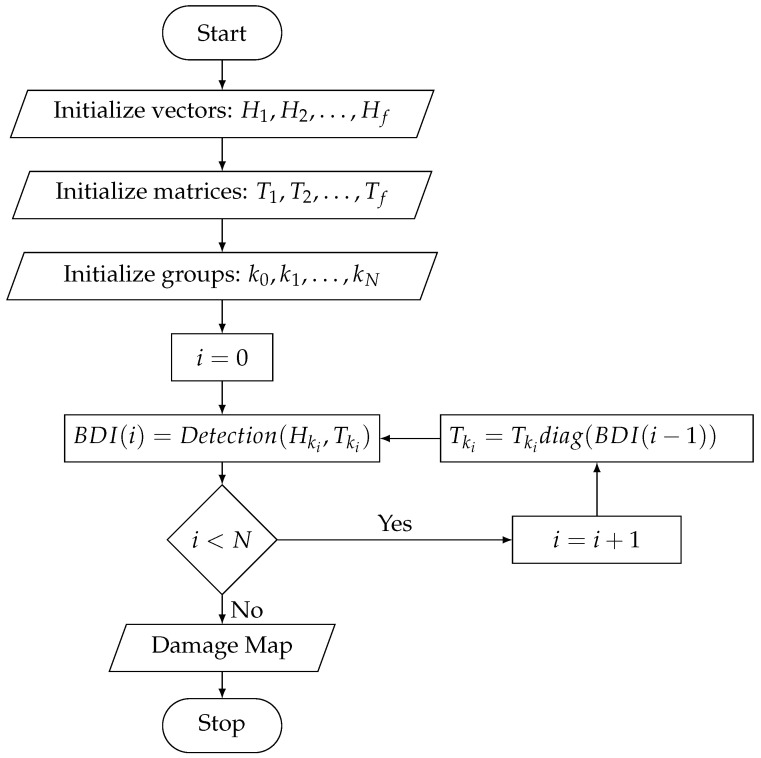
Flowchart of the Adaptive Complex Group Lasso for Damage Detection.

In this paper, an adaptive framework of Algorithm 1 is also proposed. Initial estimations for this approach are built by exploiting the broadband nature of the problem. Specifically, the frequency bins are separated into *N* arbitrary groups and a group with the corresponding regression problems is assigned as the initial one. An estimate is then obtained by applying Algorithm 1 on that group. The solution is used as a zero consistent estimate for a secondary set of regression problems on a different frequency group. For that, each of the variable vectors in the new group is pre-multiplied with the corresponding coefficient from the initial estimate. A new solution is then obtained with complex Group Lasso via Algorithm 1 on the current group. This process is iterated until all groups have been processed. The outcome from the last group is the prediction of the adaptive methodology. Algorithm 2 outlines this process in detail. A graphical representation is also given in Figure 1.
**Algorithm 2:**Adaptive Complex Group Lasso For Damage Detection
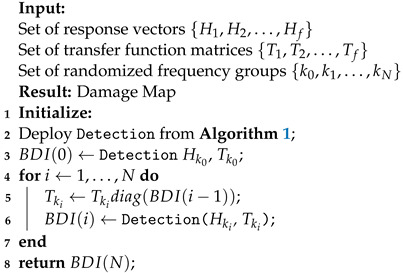


Using Algorithm 2, the prediction made for the last group of frequencies is used to plot a damage map on the structure, across the respective imaging points’ coordinates. Peaks on the map again indicate points of damage.

## 4. Experimental Validation

In this section, the performance of both routines is evaluated experimentally with goal the localization of multiple defects on a thin composite plate. The experiments make use of low-frequency acceleration responses captured with a single sensor installed on the structure.

### 4.1. Experimental Setup

**Figure 2:**
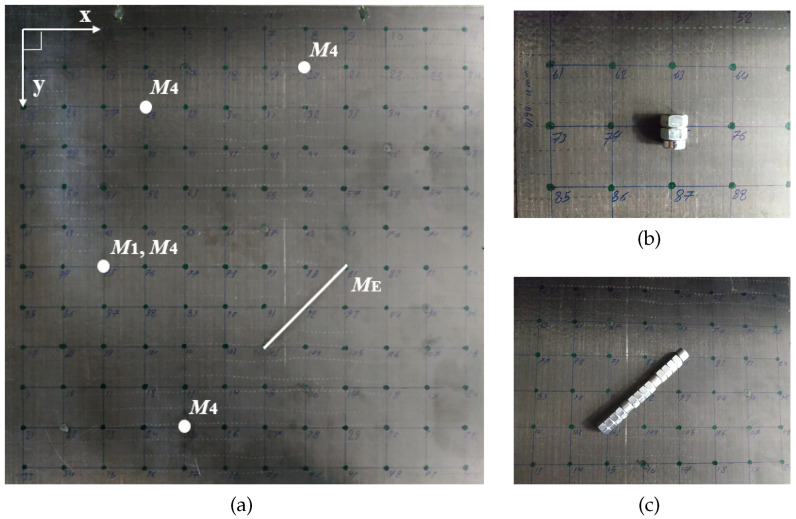
Experimental setup. (**a**) The composite plate with the selected imaging points and damage configurations $M_{1}$: 1 point-like mass, $M_{4}$: 4 point-like masses, $M_{E}$: 1 extended mass. (**b**) The implementation of a point-like mass. (**c**) The implementation of the extended mass.

A square CFRP plate with a surface of (600×600) mm${}^{2}$, cross-ply layup and 4 mm thickness was used for the experiment. Before any measurements, the set of imaging points needs to be defined on the structure. Installing a square grid of 50 mm step on the plate, defines a total of 144 imaging points as the grid’s nodes. The plate and the resulting grid are shown in Figure 2a.

Furthermore, in Figure 2a, the chosen locations of damage are also depicted. In order to simulate defects, multiple masses are installed as artificial scatterers on the healthy plate. Added masses are a simulated type of damage that differs significantly from the actual defects [40]. Nevertheless, the scattering effect they produce is suitable to represent degradation [41]. This effect alters the systems’ behavior and resembles an abrupt damage that emits information with time-invariant characteristics. Furthermore, added masses pose an ideal candidate when localization for a variety of configurations is to be studied since the baseline structure is kept intact. In total, 3 damage cases are considered in this work, presented in Figure 2a:

(i)   $M_{1}$: 1 point-like mass,
(ii)  $M_{4}$: 4 point-like masses,
(iii) $M_{E}$: 1 extended mass oriented under 45° with respect to the edges of the plate.

In Figure 2b, the implementation of the point-like masses is shown as the combination of a bolt with 2 nuts. The total mass of this configuration is 55 g and it covers a small area centered around a grid point. Figure 2c depicts the extended mass obtained as the combination of 12 nuts and 3 bolts. The total mass of this configuration is 315 g.

Opting for these scatterers enables the evaluation of the algorithm on multiple levels of complexity. Specifically, starting from a single defect we are able to progress to more complex configurations such as the case of the four point-like masses. In addition, since the baseline structure is retained intact, the case of an extended damage is also analyzed. The latter offers an additional level of complexity since such defects tend to have a more widespread effect while they also involve information about their orientation. Therefore, this additional objective must be included in the detection process.

### 4.2. Experiment

To localize the scatterers, 7 excitations are applied on the free-hanging plate. Each of them is induced as an impact with a roving hammer (PCB 086C03). The responses are then collected with a 1-D accelerometer (PCB 352a24) on the sensing position. The selected point ids, the coordinates for the 7 excitation positions ($E_{i}$) and the position of the sensor (*S*) are shown with respect to the coordinate system of Figure 2a in Table 1. The excitations are applied on the front-side of the structure while the sensor is installed on its back-side.

Before the experiment, the transfer functions connecting the excitation points and the set of imaging points must be characterized in the baseline state. This is a process performed once and is common for all damage cases. After this routine, a [7×1] vector of responses is defined for each imaging point per frequency bin. Collecting all vectors in a matrix forms a [7×144] matrix of transfer functions for each bin. This matrix is the equivalent of $T(f_{i})$ in Equation (1). In total, 8193 bins are analyzed within 0 Hz and 1600 Hz with a constant frequency step.

The 7 excitations are then applied on the damaged plates and the responses are recorded with the single sensor. Hence, this information is expressed as a [7×1] vector per frequency bin. Subtracting the baseline information from the latter returns the final vectors $H(f_{i})$. The overall process follows the description discussed in Section 2 to construct the regression problem of Equation (2).

### 4.3. Damage Detection with Complex Group Lasso

Hereafter, damage detection is performed based on the measured responses. These responses are processed as explained in Algorithm 1. As a result, a damage map is plotted on the structure using the imaging points’ coordinates and the output of the algorithm. For better visualization, an additional level of linear interpolation across the imaging points is performed. Peaks on the map indicate coordinates of high BDI and, therefore, a consistently high regression prediction across the frequency spectrum. The latter makes the corresponding imaging points strong candidates to be the positions of damage.

**Figure 3:**
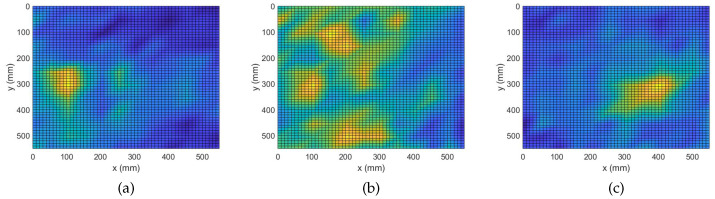
Damage Detection with Complex Group Lasso. (**a**) $M_{1}$: 1 point-like mass. (**b**) $M_{4}$: 4 point-like masses. (**c**) $M_{E}$: 1 extended mass.

During this study, a constant regularization parameter is assumed for all frequency bins of an experiment. The reason being that all broadband measurements are exposed to the same experimental conditions. To this end, assuming a value λ for the regularization parameter, k-fold cross-validation is applied on each of the broadband regression problems. Then, a cumulative error is attributed to that particular λ as the summation of the independent errors across the frequency bins. This process is iterated for a number of possible values for the regularization parameter. The value minimizing the cumulative error is selected as the optimal parameter for the experiment. Parameters equal to 0.002, 0.03 and 0.7 are, respectively, selected for the three experiments in this study. In Figure 3, results of damage localization with the complex Group Lasso are shown.

In the plots of Figure 3, regions of interest are lightly colorized peaks while valleys are shown with deep shades of blue. Figure 3a shows that the complex Group Lasso approach is able to localize the position of the single point-like mass since a unique strong peak is produced at the exact position of the defect. Hence, we can state the algorithm was successful to solve the inverse problem for one scatterer. However, when facing the more complex case of four point-like masses, it is only able to identify the signature of the defects without being able to clearly depict their exact positions. Moreover, for at least one of the masses, the indicated region spans a significant fraction of the structure’s surface. Similar behavior is expected for more and closely spaced defects. Figure 3c shows the outcome for the extended defect. Here, the algorithm was able to identify the location of interest for the extended scatterer with sufficient accuracy. Results similar to that of a single point-like scatterer were observed and the damage map demonstrated a point-like behavior while prominent information about the orientation of the damage was lost.

### 4.4. Damage Detection with Adaptive Complex Group Lasso

**Figure 4:**
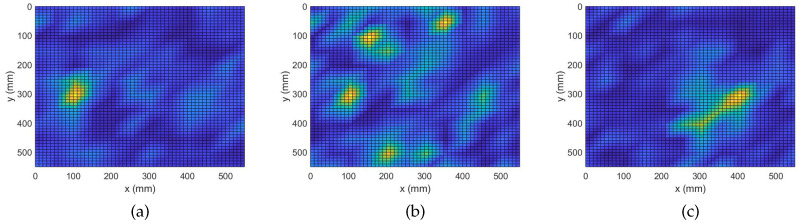
Damage Detection with Adaptive Complex Group Lasso. (**a**) $M_{1}$: 1 point-like mass. (**b**) $M_{4}$: 4 point-like masses. (**c**) $M_{E}$: 1 extended mass.

The analysis performed in the previous subsection demonstrates the ability of the complex Group Lasso methodology to identify damage signatures and localize a number of defects. It was seen, however, that in some cases, when the complexity of damage increased, the performance of the algorithm degraded. In this subsection, the adaptive approach will be evaluated on the same problem. To apply the adaptive routine with Algorithm 2, all 8193 frequency bins of the measured responses were randomly arranged into 10 groups. The obtained damage maps are shown in Figure 4.

When analyzing the graphs it is clear that all defects have been correctly localized. More precisely, it is now seen that the number of false positives has significantly decreased and the contrast between the peaks and the background is increased. Starting from the case of a single point-like mass, we already see an improvement with the center of the defect being more prominent while the area previously perceived as leakage around the peaks is significantly reduced. Next, for the case of 4 point-like masses an even greater improvement is observed. Specifically, it is seen that with the adaptive algorithm the points of damage get localized with higher accuracy, similar to that of the more trivial case in Figure 4a. Improvement is also observed for the case of the extended mass where the accuracy of localization is preserved while the secondary information about the scatterer’s orientation is recovered. Namely, it is clearly indicated that the scatterer of interest is oriented along a 45° angle with respect to the edges. Overall, the adaptive variation for the complex Group Lasso offers considerable improvement with respect to Algorithm 1.

### 4.5. Quantification and Performance Comparison

In this section, the two methods are compared quantitatively. For that, the ideal damage map needs to be considered. Such a map would introduce high peaks on the position of defects and low indications for the background. This relative difference within a damage map is used in literature to define damage zones. Yue et al. [40] apply a threshold for the top 1% of indications to form zones of detection. In this work, the relative strength of a peak with respect to the background is quantified with a Peak-to-Average Ratio (PAR). High PAR implies dominant peaks and detection within the damage map. On the other hand, an indication with PAR equal or less than 1 indicates the algorithm’s inability to resolve a defect at the given position.

**Figure 5:**
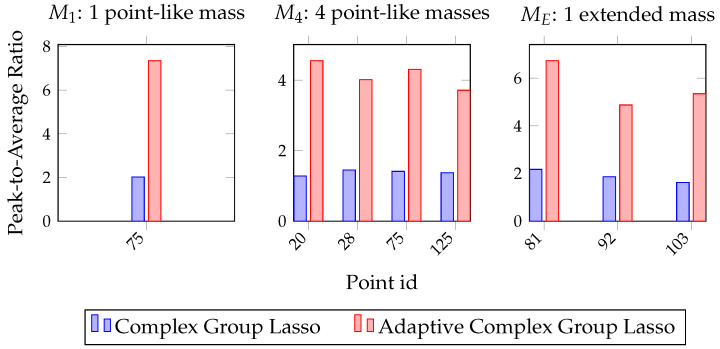
Peak-to-Average Ratio with Complex Group Lasso and Adaptive Complex Group Lasso for the three damage configurations.

As a result, a comparative metric is established that characterizes resolution and detectability on points of interest. To establish a PAR, the BDI is found for points of known degradation according to Figure 2a. Then, the result is divided by the damage map’s global average. In Figure 5, PAR values are plotted for the two methods and the three damage configurations. For the case of the single point-like mass and the four point-like masses, one and four points are characterized, respectively, while for the extended mass configuration, three points are selected across the scatterer’s geometry.

According to these graphs, the adaptive approach consistently outperforms the complex Group Lasso. Specifically, while the latter achieves peaks up to double the background average, the adaptive method returns much more dominant indications. In all scenarios, the PAR is above 1, and as also shown in the previous sections, damage is always resolved.

## 5. Performance Comparison against Full-Array Super-Resolution MUSIC

In this section, a comparative analysis is also performed for the two developed techniques against a super-resolution method of the same signal model. Specifically, the MUSIC algorithm [31] will be utilized to this end. MUSIC relies on the same signal model as the one discussed in Section 2 but extended to multi-sensor arrays. The latter is highly inefficient but offers results of extreme resolution. Those results will be used here as the reference for the damage maps produced with the Group Lasso approaches.

### 5.1. MUSIC and Super-Resolution Algorithms

Super-resolution algorithms such as MUSIC analyze overdetermined problems and form a family of methods that enhance detectability by utilizing a priori knowledge about the algebraic structure of the signals at hand. In the field of damage detection, MUSIC has been applied on structural responses to localize defects with great accuracy [42,43,44,45,46].

To apply MUSIC on the case of this paper, the underdetermined problem at hand must become overdetermined. For that, novel information is necessary. More precisely, in order for the problem of Equation (1) to become overdetermined, at least L+1 independent sources of information are needed with *L* being the number of defects. The latter, is experimentally equivalent to at least L+1 sensing elements installed on the structure, an important limitation for this method.

Due to the increased number of sensors, the vectors $H(f_{i})$ and $\varrho (f_{i})$ in Equation (1) need to be adjusted. Specifically, both vectors are to be replaced by matrices equal in columns to the number of sensors. To detect damage, MUSIC utilizes a Singular Value Decomposition of matrix $H(f_{i})$ to identify linear subspaces for the independent signal and noise information of the matrix. Assuming that the active variables span the subspace of independent information, MUSIC exploits the orthogonality those variables demonstrate with the noise subspace to produce damage maps of extremely high resolution. Such maps are usually defined through the collection of individual spectrum values across different imaging points and frequency bins. The spectrum defined at point *j* for the total of frequency bins is given in Equation (7)

(7) $$P\left(j\right)=\sum_{i=1}^{f}\frac{1}{∥T_{j}{\left(f_{i}\right)}^{*}N\left(f_{i}\right)∥},$$

where $T_{j}\left(f_{i}\right)$ denotes the *j*th column of matrix $T(f_{i})$ in Equation (1), $N(f_{i})$ is the measured noise subspace after the Singular Value Decomposition on matrix $H(f_{i})$ and ${}^{*}$ is the conjugate transpose operator.

### 5.2. Single Sensor MUSIC Damage Detection and Performance Comparison

**Figure 6:**
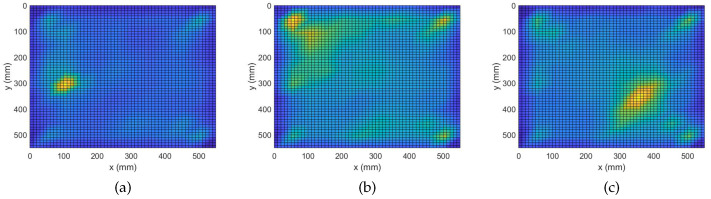
Damage Detection with Single Sensor MUSIC. (**a**) $M_{1}$: 1 point-like mass. (**b**) $M_{4}$: 4 point-like masses. (**c**) $M_{E}$: 1 extended mass.

Before introducing any novel information in the system with additional sensors, we evaluate the performance of MUSIC using the same amount of information that was available for the complex Group Lasso and adaptive complex Group Lasso. Namely, utilizing the same signals from the 7 excitations and the single sensor, the damage maps of Figure 6 are produced for the three damage cases.

In Figure 6a, the single point-like mass is successfully localized with precision similar to that of the probabilistic approaches. At first glance this may seem counter-intuitive, however, since the number of scatterers is equal to the number of sensors the system is in-fact fully determined. That is the reason the MUSIC algorithm performs with high accuracy in this case. On the other hand, when increasing the number of individual scatterers such as in Figure 6b, the system becomes ill-posed and MUSIC is not able to resolve any of the defects. The overall performance is significantly worse with respect to both Group Lasso approaches which are able to identify the damage. Furthermore, when detecting an extended scatterer, a combined effect is observed. Namely, the algorithm identifies a single equivalent point at the center of the scatterer. This approximation also produces areas of false identification at the edges of the plate.

In general, it is seen that the single sensor deterministic approach with MUSIC is only limited to cases of a single scatterer or cases of a single equivalent scatterer. When testing against more complex configurations with numerous scatterers the true limitations of MUSIC and the superiority of the probabilistic Group Lasso approaches are revealed.

### 5.3. Full Array MUSIC Damage Detection and Performance Comparison

Finally, we also perform a comparison against a full-array MUSIC. To do so, we implement the algorithm’s overdeterministic path by introducing a sensing network of 7 1-D accelerometers along the 7 excitations. Specifically, 7 sensors were installed on the positions originally given in Table 1 as excitations. As a result, on each of the 7 points of Table 1 the plate responses are simultaneously excited and recorded.

**Figure 7:**
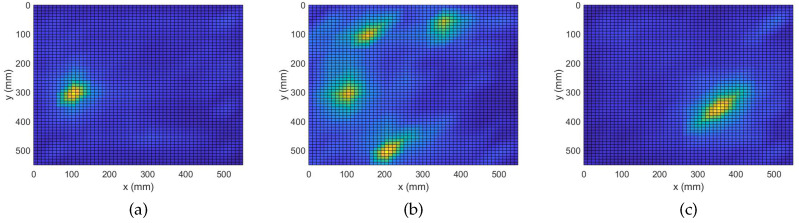
Damage Detection with Full Array MUSIC. (**a**) $M_{1}$: 1 point-like mass. (**b**) $M_{4}$: 4 point-like masses. (**c**) $M_{E}$: 1 extended mass.

In Figure 7, the MUSIC damage maps for the three configurations are shown. To produce these graphs, the same set of frequency bins, excitation signals and sensing devices were used as the ones for Figure 3 and Figure 4. At first, one may notice that for all three cases the defects were resolved with extreme accuracy. More precisely, the reduction in the number and scale of false positives is significant when compared to that of the previous graphs while all defects are highlighted with sharps peaks. It is hence safe to assume that the full array MUSIC algorithm, although inefficient, serves as a good baseline for the probabilistic complex Group Lasso and adaptive complex Group Lasso to be compared with.

In that regard, all three algorithms were able to identify with significant accuracy the position of the single point-like mass. However, it is important to notice that the adaptive complex Group Lasso ranks closer to MUSIC by excluding an area of false-positives around the defect. This effect becomes more prominent in the case of four point-like masses. For that configuration, it was already seen that the adaptive version outperformed the approach of Algorithm 1 in localizing the defects. However, it becomes even more noteworthy how well the technique ranks against the full-array MUSIC. Specifically, with both approaches the damage maps exhibit four distinct peaks, something not seen with the first technique. However, the adaptive routine achieves that with a single sensor. Finally, in the case of an extended scatterer a similar behavior between the full array MUSIC and the single sensor MUSIC is observed, whereas the adaptive algorithm brings to the foreground novel information about the damage orientation. Therefore, the adaptive complex Group Lasso contributes more meaningful information than MUSIC for the case of the extended scatterer.

**Figure 8:**
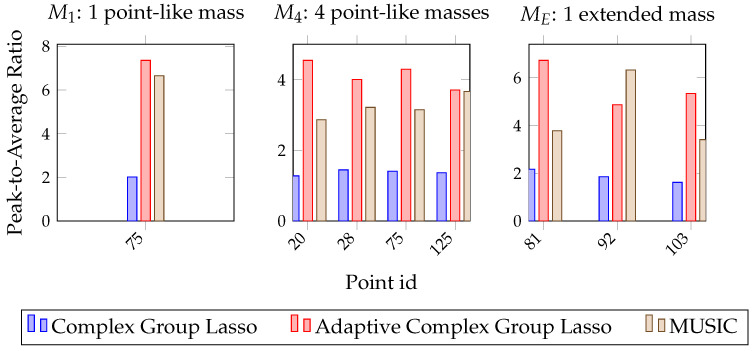
Peak-to-Average Ratio with Complex Group Lasso, Adaptive Complex Group Lasso and full-array MUSIC for the three damage configurations.

The same conclusions are drawn from Figure 8 with respect to the PAR value. In these graphs, the performance of the two algorithms is compared to that of the full-array MUSIC. Specifically, although it was already seen that the adaptive complex Group Lasso outperforms the complex Group Lasso, here it also becomes clear that the adaptive method directly competes with the full-array MUSIC. Namely, for the single point-like mass the two methods manage to achieve equivalent performance. This is also confirmed by the damage maps where the scatterer was localized as a strong indication for both adaptive complex Group Lasso and full-array MUSIC. Moreover, in the second damage case of the four point-like masses, similar behavior is observed. There, all defects are localized with both techniques while the adaptive approach leads to more prominent peaks with respect to the background. Lastly, for the extended mass configuration, both MUSIC and the adaptive method resolve at least one point across the geometry with high enough accuracy for localization. Therefore, the defects are consistently localized with both techniques. Nevertheless, the adaptive complex Group Lasso manages these results with a much smaller array.

## 6. Influence of Noise

In this section, both algorithms are evaluated under the presence of noise, a situation met in real-life applications. Specifically, when noise is present, damage detection methods must operate within the bounds of uncertainty. Throughout this paper, the effect of noise was limited since the measurements were performed in laboratory conditions, isolated from any operational or environmental parameters. In addition, the standard practice of averaging the responses was applied to further reduce noise.

**Figure 9:**
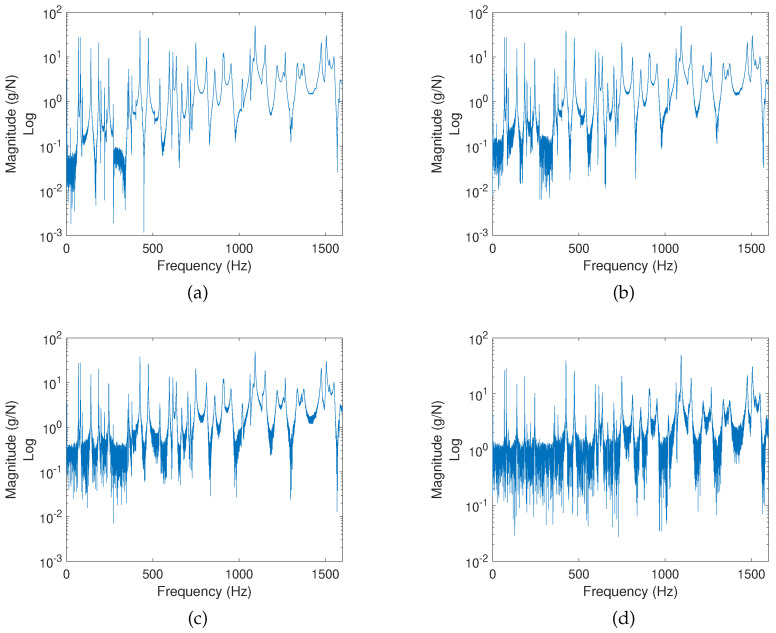
Noise-polluted FRFs for damage configuration $M_{1}$ and excitation $E_{1}$. (**a**) SNR: 40 db. (**b**) SNR: 30 db. (**c**) SNR: 20 db. (**d**) SNR: 10 db.

Thus, in the following, to quantify the impact of noise on both algorithms, different levels of additive zero-mean uniform white noise are applied to the measured responses. White noise is a special type of noise where power is evenly distributed across the frequency spectrum. In this section, different levels of noise are induced to the damaged structure’s responses to achieve predefined levels of Signal-to-Noise Ratio (SNR), varying from 40 db to 10 db. The higher the SNR the less polluted a signal is. As a result, detection with high SNR should not deviate significantly from the previous predictions. Figure 9 shows the FRF observed after an excitation at position $E_{1}$ for the damage configuration of a single point-like mass as the noise level increases. It is indeed seen that an SNR of 40 db resembles an almost noiseless case while an SNR of 10 db significantly deteriorates any meaningful information.

**Figure 10:**
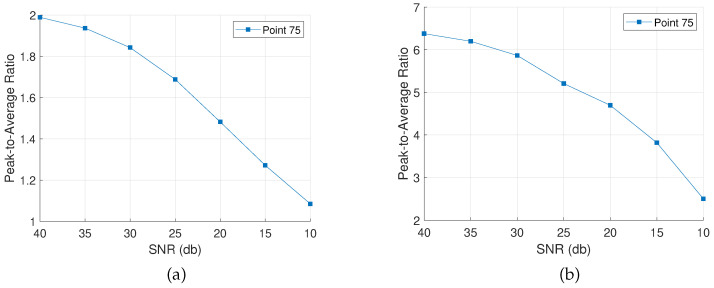
Peak-to-Average Ratio for damage configuration $M_{1}$ with varying SNR. (**a**) Complex Group Lasso. (**b**) Adaptive Complex Group Lasso.

**Figure 11:**
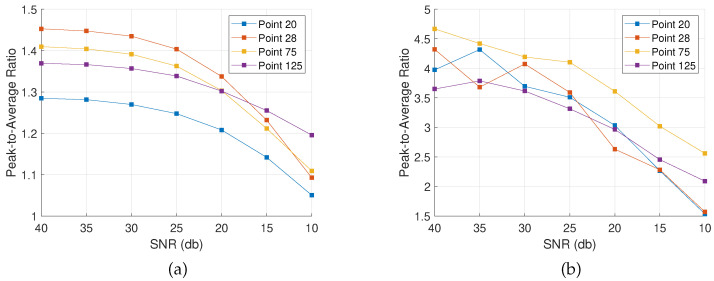
Peak-to-Average Ratio for damage configuration $M_{4}$ with varying SNR. (**a**) Complex Group Lasso. (**b**) Adaptive Complex Group Lasso.

**Figure 12:**
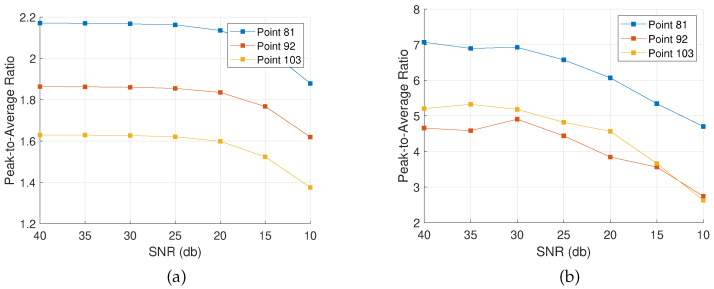
Peak-to-Average Ratio for damage configuration $M_{E}$ with varying SNR. (**a**) Complex Group Lasso. (**b**) Adaptive Complex Group Lasso.

Accordingly, noise is added to the rest of the measurements in order to evaluate the algorithms’ performance. The assessment is based on the PAR value as defined in Section 4.5 for the points of interest and the event of a successful localization. Figure 10, Figure 11 and Figure 12 show the evolution of the PAR values with increasing levels of noise for the three different configurations, applying the complex Group Lasso and the adaptive complex Group Lasso, respectively.

Each line on the graphs tracks the evolution of the PAR at a specific damage location, defined in Figure 2a. Hence, in Figure 10 a single PAR value is tracked, while in Figure 11 the PAR values at the four damage locations are analyzed. In Figure 12, the case of the extended scatterer is analyzed and three lines give the PAR values at the three grid points corresponding to the defect.

**Figure 13:**
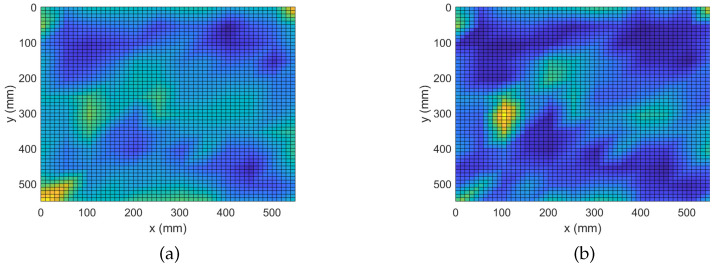
Damage Detection for $M_{1}$: 1 point-like mass and SNR of 15 db. (**a**) Complex Group Lasso. (**b**) Adaptive Complex Group Lasso.

Overall, both algorithms achieve detection for the majority of SNRs while higher levels of noise lead to lower PAR values, implying weaker indications. Moreover, the adaptive approach consistently ranks better than the complex Group Lasso, regardless of the level of noise applied. As a result, there are cases of low SNR values for which the adaptive approach is the only one resolving the defects. Specifically, while the adaptive complex Group Lasso resolves both the $M_{1}$ and $M_{4}$ configurations even for SNR close to 15 db, the performance of the complex Group Lasso has significantly deteriorated for such noise levels. This is also shown in Figure 13 where for an SNR of 15 db the adaptive approach has localized the target while Algorithm 1 has returned only a faint indication. Similar results are obtained for the other configurations.

As a consequence, for noisy environments the adaptive complex Group Lasso is preferred over the standard approach. On the other hand, for moderate to low noise levels, both techniques achieve PAR close to the one described in Figure 5 and they remain effective with well-defined peaks as discussed in the previous sections.

## 7. Conclusions

In this paper, two methodologies to perform damage detection using low-frequency complex vibration responses and compressive sensing were discussed. The first approach relied on a complex Group Lasso technique and the $l_{2,1}$-norm penalty to localize defects. The second approach introduced an adaptive logic which assumes the solutions obtained from a subset of the frequency range to serve as a good initial estimate for the solutions of another group. Both methodologies were experimentally validated for the damage detection of three defect configurations on a composite plate. Specifically, the cases of a point-like mass, four point-like masses and an extended mass were analyzed.

To this end, the complex Group Lasso was shown to be able to localize damage signatures of the defects in all three cases. Respectively, the algorithm managed to localize with high accuracy a point-like mass, as well as an extended defect. However, as the complexity of the problem arose for the case of four point-like masses, larger zones of false-positive indications were included in the solution, something that was perceived as degraded resolution. On the other hand, the adaptive complex Group Lasso, which allows independent regularization coefficients for each variable, resulted in more accurate models. Experimentally this was revealed in every case as damage maps of sharper peaks on the position of the masses and an overall lower background spectrum. Particularly for the case of multiple defects, the resolution significantly improved with respect to the first approach while for the case of the extended mass its orientation was also recovered. These results are also confirmed quantitatively by introducing a Peak-to-Average Ratio at the damage locations.

Both methods were also compared against a single sensor and a full-array MUSIC algorithm of the same signal model. There, it was seen that the two methodologies outperformed the single sensor MUSIC implementation especially in the case of four point-like masses. Moreover, when comparing against the full-array algorithm, it was seen that the adaptive approach with a single sensor performs significantly well against the reference. Specifically, for the discussed case of four defects, the methodology was able to localize the scatterers with very competitive performance, therefore offering a significantly more efficient alternative for damage detection.

The effect of uncertainty was also investigated by polluting measurements of the damaged state with different levels of white noise. Both techniques were seen to retain their effectiveness for cases of moderate to high SNR. However, as the noise levels increased, the complex Group Lasso technique deteriorated faster leaving the adaptive approach as the most suitable method for extremely noisy environments.

Lastly, as points of interest for future work and improvement, the authors suggest the use of numerical models to alleviate the need for a physical baseline. In addition, recent research has provided solutions for non-contact measurement devices such as microphones [46], high-speed cameras [47], and laser-vibrometers [48]. Such devices can be used instead of accelerometers considering they don’t require physical access to the structure. Finally, dedicated sparsity solvers can be employed to promote denoising and enhance resolution [49].

## Figures and Tables

**Table 1:** Excitation and Sensing Positions.

	$E_{1}$	$E_{2}$	$E_{3}$	$E_{4}$	$E_{5}$	$E_{6}$	$E_{7}$	*S*
Point	17	46	51	67	95	116	122	67
*x* (mm)	200	450	100	300	500	350	50	300
*y* (mm)	50	150	200	250	350	450	500	250

## Data Availability

The data presented in this study are available on request from the corresponding author. The data are not publicly available due to privacy restrictions.

## Abbreviations

The following abbreviations are used in this manuscript:

FRF	Frequency Response Function
Lasso	Least absolute shrinkage and selection operator
MUSIC	Multiple Signal Classification
BDI	Broadband Damage Indicator
PAR	Peak-to-Average Ratio
SNR	Signal to Noise Ratio
